# The need for a standard for informed consent for collection of human fetal material

**DOI:** 10.1016/j.stemcr.2022.05.013

**Published:** 2022-06-14

**Authors:** Roger A. Barker, Gerard J. Boer, Elena Cattaneo, R. Alta Charo, Susana M. Chuva de Sousa Lopes, Yali Cong, Misao Fujita, Steven Goldman, Göran Hermerén, Insoo Hyun, Steven Lisgo, Anne E. Rosser, Eric Anthony, Olle Lindvall

**Affiliations:** 1 University of Cambridge, Cambridge CB2 1TN, UK; 2 Netherlands Institute of Neuroscience (Retired), 1105BA Amsterdam, the Netherlands; 3 University of Milan, 20122 Milano, Italy; 4 University of Wisconsin-Madison/Nuclear Threat Initiative, Madison, WI, USA; 5 Leiden University Medical Center, 2333 ZA Leiden, the Netherlands; 6 Peking University, Haidian District, Beijing 100871, China; 7 Kyoto University, Kyoto 606-8501, Japan; 8 University of Rochester Medical Center, Rochester, NY 14642, USA; 9 Lund University, Lund, Sweden; 10 Harvard Medical School, Boston, MA 02115, USA; 11 Biosciences Institute, Newcastle University, Newcastle upon Tyne, UK; 12 Cardiff University, Cardiff CF10 3AT, UK; 13 International Society for Stem Cell Research, Skokie, IL, USA

## Abstract

The ISSCR has developed the Informed Consent Standards for Human Fetal Tissue Donation and Research to promote uniformity and transparency in tissue donation and collection. This standard is designed to assist those working with and overseeing the regulation of such tissue and reassure the wider community and public.

## Main text

The research collection of human fetal material from the termination of pregnancies involves complex ethical and logistical issues, and the processes for doing this vary widely from country to country. As a result, tissue collected at one site may not be allowed to be used at another that operates under a different regulatory framework and ethical perspectives. In order to address such issues and to ensure that this vital source of material can be used to the maximum benefit of basic and clinical science and therapeutics, the ISSCR convened a taskforce of relevant experts to draft a common informed-consent standard for the donation of human fetal tissue (https://www.isscr.org/docs/default-source/policy-documents/isscr_informedconsentstandardforhumanfetaltissuedonationforresearch__4-20-22_final.pdf).

Human fetal material has proven a vital source of tissue for many different areas of research over the last 30–40 years. Research using such tissue has expanded of late as the technologies for analyzing tissue at the single-cell level with spatial transcriptomics and proteomics have opened up new ways of understanding normal human development (Haniffa et al., 2021; Park et al., 2020; Bocchi et al., 2021) as well as the origin of certain cancers (Young et al., 2021; Kildisiute et al., 2021) and developmental/congenital abnormalities (Samad and Wu, 2021). In addition, the translation of stem cell interventions to first-in-human trials has meant that there is a need, in some cases, to assess the fidelity of that stem cell product against its developmental equivalent (Grealish et al., 2014). The request for human fetal material collected through termination of pregnancies is rising at a time when the ability to collect fetal tissue is becoming more challenging, as such procedures are increasingly being moved into the community through medical, rather than surgical, approaches. This has created a need to better harmonize the consent for the collection and distribution of this tissue while being cognizant of the different ethical and regulatory frameworks that exist in different countries.

Human fetal material has unique properties that cannot be replicated using other tissue sources. Recently, work has shown that certain populations of cells found in the normal developing human fetus are simply not seen in rodent models of development (Park et al., 2020; La Manno et al., 2016), and thus without the study of such tissue, important cell types and developmental pathways would be missed. Furthermore, this tissue has been used to understand diseases that preferentially target developing human tissues such as the Zika virus (Retallack et al., 2016). Finally, this tissue has been used for proof-of-principle trials, for example, in patients with Parkinson’s disease (PD) in whom fetal midbrain tissue implants were used, with some successes, to replace lost midbrain dopamine neurons (Bjorklund and Lindvall, 2017).

In this new ISSCR document, we have sought to establish an international standard for informed consent that will promote uniformity and transparency in tissue donation and collection. Such a standard should serve to assist those working with the tissue and those overseeing the regulation of the use of such tissue as well as reassure the wider community and public.

The standard covers what was deemed to be the major areas of this consenting process but should be viewed in the context of national laws and guidelines that already exist for such donations. The areas laid out in the standard cannot cover all eventualities and the type of research using human fetal tissue, as this has its own guidelines and is not strictly relevant to the consenting process itself. Nevertheless, a necessary part of the information shared with the donating women is the need to know what potential uses may be made of the tissue so donated.

The key issues that should be covered include:

• The separation of the consent for termination of pregnancy from consent for the potential use of the fetal material, such that the latter consent should only be sought after the consent for the termination of the pregnancy has been obtained. This is to ensure that the women terminating the pregnancy does so without any sense of coercion or encouragement to proceed, knowing she is donating the tissue for some research/therapeutic purpose. Indeed, it is critical that the donating woman understands that the termination and potential use of the tissue are separate consents and that both are done in a voluntary fashion and that either consent can be withdrawn at any time up to the point of tissue collection. Such a decision will not impact on their medical care, nor does it mean that giving consent for the donation of tissue changes in any way the standard of medical care they will receive.
• The decision to donate tissue does bring with it, in some instances, additional tests, which need discussing with the donor woman, as some of these may have implications for them. For example, additional blood tests to look for certain infections may reveal that the donating woman has an infection she was unaware of and that this has important consequences to them and others (e.g., HIV infection, syphilis, and so on). Given this, a clear process for dealing with such rare results needs to be established in any fetal-tissue donation program.
• Potential use of the tissue is an important area to be discussed. Although a complete list of uses for any tissue is not possible, given how techniques and questions evolve in clinical science, the list of ongoing work and likely new work should be discussed. The level of detail may vary, but it should at the very least describe what is being done with the tissue and why, how the research is funded, and what potential benefits may arise from the work as well as how the research project has been reviewed and approved. In addition, any conflicts of interest (both financial and research) should also be declared to ensure that the work is being done in a transparent way. This is particularly relevant when the tissue is being used for the development of potential commercial products. Also, issues around confidentiality of the donor need to be covered as well as what will ultimately happen to the tissue in terms of storage and/or disposal.
• Finally, at the end of the consenting procedure, there should be an opportunity, including, if needed, an adequate but realistic time period (e.g., a few days) for the woman donating the tissue to ask any questions of the team obtaining the consent and/or providing links/names of those who may be able to provide more information on particular aspects of the fetal-tissue research. However, it should be stressed that ideally (and in some countries, this is covered by regulation) those collecting the consent for the tissue are not the same people as those who will be undertaking research with it so as to ensure there are no perceived conflicts of interest and/or coercion.

In conclusion, this new standard has been developed to help all working in this area of research with the hope that it will facilitate such work with this important tissue source. This standard is not seeking to offer an opinion on the ethical and legal rights of termination of pregnancy or fetal-tissue research, which are covered in other guideline documents from the ISSCR (https://www.isscr.org/policy/guidelines-for-stem-cell-research-and-clinical-translation).
